# Exploring Bidirectionality in Global Health Partnerships: Perspectives From U.S. Pediatric Residency Programs

**DOI:** 10.7759/cureus.108918

**Published:** 2026-05-15

**Authors:** Anik Patel, Pauline Kamau, Justus Simba, Ashley Combs, Adelaide Barnes, Kathy Ferrer, Heather Haq, Duncan K Hau, Lee Morris, Brittany Murray, Amy Rule, Reena P Tam, Lisa Umphrey, Megan S McHenry

**Affiliations:** 1 Pediatrics, Children's Mercy Hospital, Kansas City, USA; 2 Pediatrics, Indiana University School of Medicine, Indianapolis, USA; 3 Pediatrics, Jomo Kenyatta University of Agriculture and Technology, Juja, KEN; 4 Pediatrics, Children's Hospital of Philadelphia, Philadelphia, USA; 5 Pediatrics, Children's National Hospital, Washington, D.C., USA; 6 Pediatrics, Baylor College of Medicine, Houston, USA; 7 Pediatrics, Weill-Cornell Medical College, New York City, USA; 8 Pediatrics, Atrium Health, Charlotte, USA; 9 Pediatrics, Emory University School of Medicine, Atlanta, USA; 10 Pediatrics, University of Utah Health, Salt Lake City, USA; 11 Pediatrics, Children's Hospital Colorado, Denver, USA

**Keywords:** academic partnerships, bidirectionality, global equity, global health education, pediatric residency

## Abstract

Introduction

Despite growing interest, little guidance exists to re-evaluate global health partnerships (GHPs) through promoting bidirectionality. This study aims to understand the perspectives of United States (U.S.) pediatric global health (GH) faculty on institutional bidirectional practices to inform future discussions with international collaborators.

Methods

An internally, iteratively developed REDCap-based online survey (REDCap, Vanderbilt University, Nashville, TN), by our diverse author group, collected qualitative data from January 18 to February 20, 2023. Free-text responses were reviewed and coded inductively, with findings framed to generate themes for subsequent engagement with international partners.

Results

Of the 88 U.S. programs that identify as having GH programming, participants from 22 programs responded, of which most (n=18) were “very familiar” with bidirectionality, described in terms of reciprocity, equity, and collaboration. Perceived benefits of bidirectionality included friendship and mutual respect, educational opportunities, and opportunities to achieve equity. Perceived challenges included technical barriers, cultural barriers, mismatched expectations, and financial barriers. Despite challenges, participants expressed that persistence and flexibility from U.S. institutions are necessary to create and maintain deeper, partner-informed collaboration through bidirectional GHPs.

Conclusions

Our results from U.S. perspectives suggest that bidirectionality could be defined as a concept whereby two or more partners collaborate at the personal and institutional level, resulting in reciprocal and equitable exchange of benefits. By first examining U.S. pediatric perspectives, this study provides a reflective framework to support future engagement with international partners in assessing bidirectionality in their current GHPs. Establishing bidirectionality in GHPs may look different across settings, but the mutual benefits, frequent reflection, and evaluations by both partners are necessary.

## Introduction

Global health is defined as “the study, research, and practice that prioritizes achieving equity in health for all people” [1]. Over the last two decades, interest in global health among medical trainees in the United States (U.S.) has increased [2,3], which has led to an expansion of global health training opportunities across the U.S. [4]. Many of these programs include immersive experiences at international partner sites through established academic global health partnerships (GHPs). As these partnerships have become more common, greater attention has been directed toward how they are structured, implemented, and sustained. Examining how U.S. institutions conceptualize and operationalize GHPs offers important insight into how to promote more meaningful and equitable engagement with international colleagues.

GHPs between pediatric institutions in high-income countries (HIC) and low- and middle-income countries (LMIC) settings can provide mutual benefits in medical education and faculty development, although the nature and extent of these benefits may differ across partners and contexts. Pediatric trainees from HICs often gain knowledge and clinical experience working in resource-constrained settings, strengthen physical examination and procedural skills, develop greater awareness of cultural and socioeconomic determinants of health, and learn to navigate healthcare systems distinct from their own [5-7]. In contrast, pediatric trainees from LMIC partner sites who participate in exchanges to U.S.-based sites report the development of enhanced teaching and feedback skills, improved ability to interpret medical literature, increased research competencies, and expanded clinical knowledge (including subspecialty clinical knowledge), and awareness of resources [2,8,9].

For many trainees within HIC medical institutions, the opportunity to travel to and learn from colleagues from LMICs is a highlight of their training. However, U.S. pediatric trainees immersed in LMIC partner sites require ongoing support to navigate logistical, clinical, language, and cultural challenges, which may place an additional burden on faculty, staff, and trainees at the LMIC site [10]. U.S. pediatric programs do not commonly host trainees from LMIC partner sites for reasons that often center around privilege and power, such as deprioritized travel funding, medical licensing requirements, and national immigration policies [11]. Due to restrictive medical licensing options, exceedingly few U.S. programs are able to provide patient-facing opportunities for visiting trainees from LMIC [12], whereas U.S. trainees often have hands-on clinical experiences while at LMIC partner sites [13]. Despite these challenges, GHPs can be the cornerstone for advancing research, public health, clinical work, advocacy, and education for all trainees and faculty involved [10,14].

Historically, global health medicine was designed to uphold the colonial economies and protect the health of HIC countries’ personnel rather than benefit local populations [15]. Relationships between academic pediatric institutions from HIC and LMIC settings are often fraught with economic and political power imbalances that continue to perpetuate historic colonialism paradigms [16-18]. In recent years, these relationships have been re-evaluated through the lens of equity and decolonization, which is defined as the concept of “identifying, unveiling, and interrupting the persisting remnants of colonialism” in medicine and social structures [15]. For GHPs committed to decolonization within the partnership, more attention must be given to developing ongoing, shared reflection and dialogue among all partners regarding the educational experiences and career development of trainees and faculty. Critically, this process requires intentional reflection by U.S. institutions on their own assumptions, practices, and definitions of bidirectionality when engaging with international partners.

Ensuring GHPs are thoughtfully developed from the perspectives of both HIC and LMIC sites is imperative to creating mutually respectful, equitable, and beneficial partnerships [10,14]. With growing interest in bidirectional exchanges [2,10], we sought to examine perspectives from U.S. academic pediatric global health programs as one situated lens through which to reflect on the concept of “bidirectionality.” As an initial step towards engaging with both U.S. and international partner perspectives, first, we explore U.S. global health faculty perspectives on their own institution’s practice of bidirectional activities, including perceived benefits and challenges. These findings will provide insights to guide further discussion with international collaborators regarding their own perspectives for bidirectional engagement, and to contribute towards efforts to decolonize global health.

## Materials and methods

Setting and participants

We performed a cross-sectional survey from January 18, 2023, to February 20, 2023, examining GHP bidirectionality in U.S. pediatric residency programs. We contacted all U.S. pediatric residency programs accredited by the Association of American Medical Colleges (AAMC) via email to describe the study objectives and to seek contact information for faculty or staff responsible for global health education within their residency program. We then emailed each faculty or staff member to confirm interest in study participation. During this first phase of the project to explore bidirectionality from partner institutions, inclusion criteria were faculty or staff at U.S. pediatric residency programs accredited by the AAMC and involved, at any level, in pediatric resident global health education. Faculty or staff at U.S. pediatric residency programs with no global health education programs available for residents were excluded from the study. Only one study participant per program was invited to participate. The respective university’s Institutional Review Board determined this to be an exempt study.

Survey development and data collection

Our author group, representing multiple types of GHPs including U.S. pediatric academic institutions and partners from LMIC settings, internally developed questions for an online, REDCap-based survey using an iterative process. As the survey was a product of our author group, no permissions or license agreements were required to distribute and publish our survey. Our survey questions focused on demographic, fixed-choice, and open-ended items on topics related to GHPs and bidirectional exchange. Response options included Likert scales, free responses, or multiple-choice answers (see Appendix 1 for survey). The surveys were iteratively developed within our diverse author group with U.S. respondents in mind, with the team's intent to adapt the survey for global colleagues as guided by the initial insights from U.S. faculty. We distributed REDCap survey links to potential participants via email (Appendix 2). Participants earned a $5 gift card as remuneration for their time.

Data analysis

We calculated descriptive statistics for all variables. We evaluated Likert scales as ordinal numbers and summarized results with means and standard deviations. We reviewed and performed line-by-line coding of free-text responses through an inductive approach by 3 independent researchers with experience in qualitative analysis. We used an a priori framework to organize qualitative data into the following domains: (1) Defining “Bidirectionality”, (2) Perceived Benefits of Bidirectionality, (3) Perceived Challenges of Bidirectionality, and (4) Strategies for Overcoming Barriers. Using constant comparison, the listed researchers identified emerging themes that they then triangulated to identify central concepts. A fourth coder was available for instances where consensus was not reached. 

## Results

Participant and program characteristics

**Figure 1:**
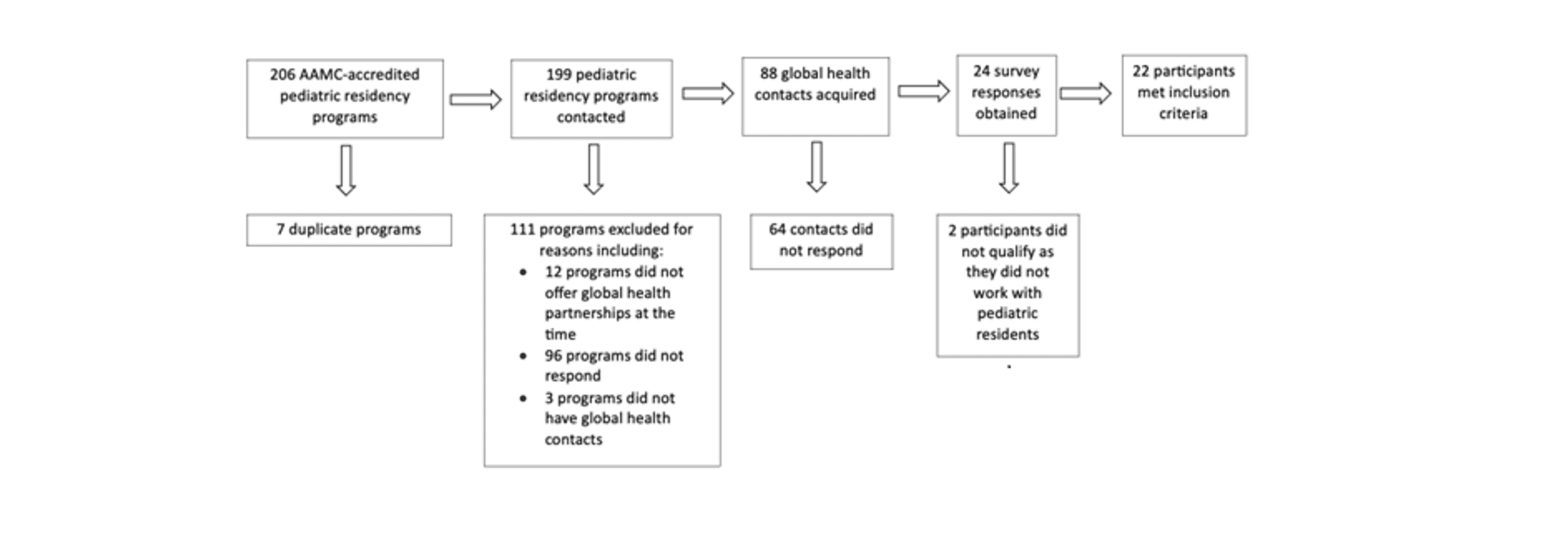
Methodology for inclusion and exclusion of potential participants

Of the 206 AAMC pediatric programs, seven programs appeared to be at the same institutions; thus, 199 AAMC-accredited individual pediatric residency programs were contacted. Of these 199, 88 responded with contact information for GH faculty or staff. There were 24 survey respondents (27% response rate), with two participants’ responses excluded as they did not work with pediatric residents (Figure 1).

Of these 22 respondents, 15 were pediatric GH program directors (68%), three were faculty who work with pediatric residents (14%), and four self-described as the director of all GHPs (not just pediatrics), GH educator, or program manager/coordinator. Participants came from pediatric programs of varying sizes, with six having 1-30 residents (6/22, 27%), eight having 31-60 residents (8/22, 36%), one having 61-90 residents (1/22, 5%), six having more than 90 residents (6/22, 27%), and one participant was uncertain (1/22, 5%). Additionally, programs varied in the number of residents with expressed interest in global health at any given time, with 10 having 1-10 GH residents (10/22, 45%), six having 11-20 (6/22, 27%), two having 21-30 (2/22, 9%), and four participants unsure (4/22, 18%). Around 95% (21/22) reported that their pediatric residency programs offered GH electives, which included virtual (5/22, 23%), local community (12/22, 55%), domestic (U.S.-based but not local) (9/22, 41%), or international (20/22, 91%) experiences. Nearly three-quarters of programs had formalized GH tracks (15/22, 68%) or formalized GHPs with international sites (16/22, 73%). Over half of participants were between the ages of 36 and 45 (13/22, 59%), and had variable durations of GH experience, with several reporting 11-15 years of experience (9/22, 41%). Just over half of participants identified as female (12/22, 55%) and less than a third of participants identified as non-White/Caucasian (7/22, 32%) (Table 1).

**Table 1:** Demographic characteristics of participants

Participant Characteristics	n/22 (%)
Role within residency program	
Global health director	15 (68%)
Faculty interested in GH	3 (14%)
Global health educator within program (but not faculty or director)	1 (5%)
Other	3 (14%)
Number of pediatric residents in program (total)	
1-30	6 (27%)
31-60	8 (36%)
61-90	1 (5%)
91+	6 (27%)
Unsure	1 (5%)
Number of pediatric residents with expressed interest in global health	
1-10	10 (45%) includes one value of 2-6 and one value of 8-12
11-20	6 (27%)
21-30	2 (9%)
Unsure	4 (18%)
Does your program offer a formal GH track?	
Yes	15 (68%)
No	6 (27%)
Unsure	1 (5%)
Does your program offer a GH elective?	
Yes	21 (95%)
No	1 (5%)
I don’t know	0 (0%)
Does your program offer a GH elective that is virtual?	
Yes	5 (23%)
No	17 (77%)
I don’t know	0 (0%)
Does your program offer a GH elective that is locally-based?	
Yes	12 (55%)
No	10 (45%)
I don’t know	0 (0%)
Does your program offer a GH elective that is domestically-based?	
Yes	9 (41%)
No	13 (59%)
I don’t know	0 (0%)
Does your program offer a GH elective that is internationally-based?	
Yes	20 (91%)
No	2 (9%)
I don’t know	0 (0%)
Does your program offer a GH elective that is not described by the previous categories?	
Yes	0 (0%)
No	22 (100%)
I don’t know	0 (0%)
Prior to the COVID-19 pandemic, how many pediatric residents did a domestic or international GH elective?	
0	2 (9%)
1-5	8 (includes a value of 3-6) (36%)
6-10	4 (18%)
11-15	5 (23%)
16-20	0 (0%)
21-25	1 (5%)
I don’t know	2 (9%)
Does your pediatric residency program have any GH partnership sites?	
Yes	16 (73%)
No	4 (18%)
I don’t know	2 (9%)
How familiar are you with the term “bidirectionality”?	
5 (extremely familiar)	0 (0%)
4 (very familiar)	18 (82%)
3 (familiar)	3 (14%)
2 (somewhat familiar)	0 (0%)
1 (not familiar at all)	1 (5%)
Do you believe your pediatric program is engaging in bidirectionality?	
Yes	19 (86%)
No	3 (14%)
Age	
36-45	13 (59%)
46-55	4 (18%)
56-65	1 (5%)
66+	2 (9%)
Prefer not to answer	1 (5%)
Race (select all that apply)	
Asian	5 (23%)
Hispanic/Latinx	2 (9%)
Native Hawaiian/Pacific Islander	0 (0%)
African-American/Black	0 (0%)
White	15 (68%)
Native American	0 (0%)
Prefer to not answer	1 (5%)
Ethnicity (Hispanic/Latinx)	
Yes	2 (9%)
No	18 (82%)
Prefer to not answer	2 (9%)
Indigenous	
Yes	0 (0%)
No	21 (95%)
Prefer to not answer	1 (5%)
Gender	
Male	9 (41%)
Female	12 (55%)
Prefer to not answer	1 (5%)
Years of GH experience	
1-5	3 (14%)
6-10	3 (14%)
11-15	9 (41%)
16-20	2 (9%)
21-25	3 (14%)
26+	1 (5%)
Prefer to not answer	1 (5%)

Domain: defining bidirectionality

Eighteen participants (82%) stated they were very familiar with the term “bidirectionality”, while three felt familiar with the term, and one did not feel familiar at all with the term. Nineteen (86%) participants stated their programs participated in bidirectionality, although the examples submitted to describe their bidirectionality structure varied. A third of participants described their structure as medical students or graduate medical trainees from LMIC settings coming to U.S. pediatric programs for visits or observerships (8/22, 36%). The next most common structures were bidirectional exchanges of medical trainees in both directions for clinical work (4/22, 18%) and only U.S. medical students, trainees, or faculty members going to GH partner sites to teach or do clinical work (2/22, 9%). Other examples included the creation of research and innovation collaboratives (5/22, 23%) or the formation of consortia or collaborations with various institutions to improve infrastructure and capacity building (2/22, 9%). Another common example cited digital or virtual educational opportunities for faculty and trainees from both sites to participate, such as morning reports, didactics, or a flipped classroom structure (whereby students encounter learning material before class and then can spend time in informed discussions) (3/22, 14%). One pediatric program offered affiliate appointments to partner faculty, while another described how their program financially supported partnering international physicians visiting other partner sites abroad to learn best practices in similar LMIC settings.

As a concept, many participants shared similar ideas about what components they believe comprise a bidirectional partnership, particularly the concepts of reciprocity, equity, and collaboration. When asked what bidirectionality means, most discussed reciprocity using words and phrases such as “win/win”, “vice versa,” “both”, and “bilateral.” Participants indicated that bidirectional partnerships involved a “give and take”, an “exchange,” resulting in “shared” or “mutual [benefits].” According to participants, such reciprocity went beyond an exchange of individuals or money to include an exchange of culture, perspectives, and knowledge.

“Partners see themselves as mutually beneficial to one another and resources such as knowledge, research, and people are exchanged and shared roughly equally between the partners.”

Half of the participants explicitly discussed that reciprocity meant equitable, equal, or similar opportunities or benefits for both parties involved. In describing bidirectionality, participants often used the words “equity,” “equitable,” “equally,” and “fairness.” Participants described striving for bidirectionality in order to “avoid colonial dynamics” and to create “power equity.” Bidirectionality was seen as a way to “diminish the [potentially] extractive nature” of a partnership between partners with disparate resources.

“To accomplish such a relationship, parties must be very aware of the power dynamics at play and seek to create greater power equity.”

Participants also described different collaboration modalities, such as teaching trainees at both sites or conducting research together. One participant expressed that bidirectional partnerships are ones where “We are invited in as guests. Local people are experts, but by combining their talents and strengths with our outside experiences, we can create effective solutions to problems community members identify.” Another description was partners working together to solve problems or toward a shared outcome. In addition, participants expressed a desire to have partnerships not just at the institutional level, but also at a personal level. Some participants used words and phrases such as “relationship,” “shared decision making,” “support one another,” and “collaboration.” Respondents indicated that they believed partners should be accountable to one another, share common goals, and be mutually invested in maintaining a relationship. 

Domain: perceived benefits of bidirectionality

Just over half of the participants emphasized that bidirectional partnerships can enhance learning. Participants described that being in new or different environments can challenge medical dogma and medical knowledge. For instance, one participant stated that bidirectionality allows for “learning in [an] unexpected way...understanding how medicine is done in [an] unfamiliar place.” It can introduce clinicians to new diseases and pathologies as reflected by a participant who stated that there is an “expansion of medical knowledge in diagnosis and treatment of diseases or disease states unfamiliar or uncommon [to us].” Bidirectional partnerships can provide new insights into one’s own medical practice, as indicated by a participant who said that these partnerships give a “better understanding of the limitations in technology. At the same time, understanding how reliant we are on tests and weakness[es] in clinical diagnosis.” According to participants, bidirectional partnerships allow for shared learning in environments such as digital or virtual morning reports or didactics, allowing partners to learn directly from each other, as reflected by a participant who stated, “While in the U.S., the Kenyan trainees work alongside [institutional] trainees and they get to learn casually in talking about clinical experiences. Kenyan residents will give a noon conference about clinical issues that routinely arise in their pediatric care.”

Beyond academic education and clinical training, participants also expressed that a new environment at another partner site can create “perspective building”, develop “cross-cultural skills building”, “build empathy,” foster gratitude, and stimulate self-reflection.

*“We have so much to learn from one another. Building relationships and seeing the world from other perspectives makes our skills as clinicians and knowledge as humans so much more rich.”* 

Some participants also stated that bidirectional partnerships provided opportunities to build mutual respect and for HIC partners to enhance LMIC partners’ “autonomy and agency.” Bidirectionality “raises the level of respect for each party.” It allowed them to “recognize both groups as being valuable partners.”

Domain: perceived challenges of bidirectionality

Despite the many perceived benefits of a bidirectional GHP, participants described a variety of challenges faced in building and developing partnerships, such as national/local travel restrictions, limitations in resources, long-distance communication with virtual partners, institutional barriers, cultural and language barriers, and navigating power dynamics. These challenges often result in LMIC partnerships having limited opportunities for travel and learning in the U.S.

Challenges Related to National/Local Travel Restrictions

Participants discussed the challenges of health-related travel restrictions, particularly in relation to the COVID-19 pandemic, which at times allowed U.S. trainees to travel to LMICs, but those trainees from LMIC partner sites were not allowed to come to the U.S. for continued learning.

Challenges Related to Limited Resources

Limited time and financial resources further restrict trainees in LMIC settings from visiting U.S.-based institutions. Most survey participants discussed issues with trainee travel finances, with words such as “funding”, “money,” “resources,” “cost,” and “expenses'' being utilized when discussing challenges to bidirectionality. Faculty or trainees visiting from LMIC settings often self-fund their own visit. When funding for creating and developing GHPs was available, participants described varied sources, including across academic departments and institutions, foundations or endowments, philanthropic donations, and non-governmental organizations. Even if visits are funded, participants described income loss for visiting faculty or trainees when coming to the U.S.

For example: *“Residents in most of Africa pay to go to residency. They have moonlighting jobs to make ends meet. So asking them to come to the U.S. for an educational opportunity means they have to forgo jobs that help pay for rent and tuition. This is a financial burden.”*

Participants often mentioned time limitations for developing, maintaining, and nurturing bidirectionality, with participants stating: “It’s time-consuming” and “It can take more time to develop these relationships.”

Challenges Related to Communication within Virtual Partnership Engagement

Some participants wrote about the logistical difficulties that come with spanning time zones. One participant stated, “Time difference has been a consistent barrier to virtual meetings and conferences,” while another stated that “time and scheduling conflicts'' have been a barrier to engaging in bidirectional partnerships. In addition to barriers related to timing, internet connectivity difficulties were brought up by some participants: “Communication over [the] internet [makes it] difficult from afar.”

Institutional Barriers

Some participants discussed the institutional barriers that hindered building bidirectional partnerships. This is further compounded when “U.S.-based institutions [are] fearful of litigation and legal exposure.” In addition to such policies, participants described challenges with perceived “lack of support at the university, school of medicine, and hospital level” and wavering “will of leadership and institutions that may not be invested in these long-term relationships.” One participant stated, “There is a ‘What’s in it for our institution?’ attitude...There is a misunderstanding of the goal of bidirectionality.” A few participants additionally brought up the “lack of administrative staff to help with coordination” and “the burden this creates for administrative staff” as barriers to building and sustaining bidirectionality. 

Local Policy Barriers 

Some participants described that partners from LMIC settings have difficulties overcoming prohibitive requirements for clinical credentials in the U.S., due to state law or institutional policy. This results in LMIC trainees and faculty often only being able to observe clinical care rather than providing direct patient care. Some GH faculty state that U.S.-based institutions [are] unable to allow visiting trainees to interact with patients.”

Challenges Related to Culture and Language 

Many participants described cultural barriers to bidirectionality, in particular when there were differing beliefs: “Differences in perspectives; lack of understanding of differing lived experiences and cultural values; [and feelings of] entitlement.” These differences can lead to “cultural clashes,'' potentially leading to “distrust when people are not communicating well.” Bridging language and cultural norms can be a significant barrier, as well; “Language [is] perhaps the largest'' barrier, stated one participant, while another stated that “language and customs can pose barriers in delivery or understanding.” One participant, in addition, brought up that LMIC faculty and trainees can experience “discrimination against outside visitors”, although the participant did not elaborate further on examples of this in the survey.

Domain: strategies for overcoming challenges

Despite the many challenges of creating and maintaining GHPs, one participant expressed that persistence was needed to overcome barriers. Another participant described finding purpose outside of financial benefit for themselves:

“There is no set aside time for me to be the track director. I do it because it’s my passion.”

Participants provided various examples of flexibility to overcome barriers. A few (2/22, 9%) participants described using ingenuity for finding funding sources from grants (“specific training grants for international research training”) or from funding sources outside of the department of pediatrics (“It is funded through the Institute for Global Health”). Some programs have overcome financial challenges related to traveling by optimizing technology to facilitate discussions and foster learning: “We have utilized virtual platforms to accomplish training/collaboration.” One participant stated that their program adjusted the timing of their didactic or meeting times to accommodate various time zones. In addition, to ensure ongoing communication, this participant’s program also “pays for data bundles.” Despite these efforts, some participants (4/22, 18%) continued to express that this was a work in progress, with one participant saying that “we have not yet [overcome these barriers]” and that they are still “actively working on it.” 

## Discussion

Engaging with U.S. institutional perspectives, in dialogue with international colleagues, may support more equitable global health partnerships. This study examines U.S. institutional perspectives not as definitive, but as one lens intended to support reciprocal dialogue with international colleagues. We examined the insights and perspectives of U.S. pediatric residency GH educators in order to better understand how U.S.-based individuals view and implement “bidirectionality” in GHPs. Within our small sample of educators, we found important considerations for U.S.-based pediatric GH educators in forming and maintaining bidirectional GHPs, which have general applicability to GH educators in other specialties, as well as international colleagues. Our U.S.-based GH educators supported the notion that bidirectionality in GHPs is a concept whereby two or more partners collaborate at the personal and institutional level, resulting in benefits that are reciprocal and equitable. In addition, bidirectional GHPs are believed to be based on mutual respect and understanding, not merely an exchange of financial or educational resources. These results create a foundation of understanding from one perspective of the partnership, with future work to be focused on understanding perspectives from LMIC academic partners.

Although GHPs are commonly understood to provide benefits particularly for trainees from HIC settings, the impact can be difficult to describe in measurable units such as time, revenue, or number of academic publications, which may contribute to the difficulty with objectively quantifying the full scope of benefits for U.S. academic programs to participate in GHPs. This challenge may be particularly pronounced in bidirectional partnerships, where the most meaningful outcomes are relational, which institutions may struggle to recognize or value within traditional academic metrics. We found the benefits of GHPs to be more abstract, such as greater cultural understanding, increased perspective building and empathy, and increased flexibility and adaptability. In contrast, challenges to establishing bidirectional GHPs were more easily quantifiable, such as limitations in time or financial resources. Situating these findings as one institutional perspective based in the U.S. highlights the need for shared approaches to defining and valuing benefits across partners. We suggest linking U.S. trainee milestones during residency to measures of personal growth, cultural humility, and clinical skills, including opportunities for U.S. participants in GHPs to reflect on their self-growth as physicians and as individuals. These efforts may also be applied to LMIC trainees in order to support U.S.-based opportunities often prohibited by cost. However, global voices from LMIC-based trainees are equally important to explore prior to implementing measurable ways to quantify the abstract benefits of equitable GHPs.

Furthermore, significant barriers and challenges, such as legal and financial barriers, can deter some U.S. pediatric programs from pursuing bidirectionality. Legal barriers such as visa difficulties or obstacles to credentialing can only be alleviated with policy changes that allow for pediatric trainees from abroad to enter the U.S. and to also participate in supervised clinical care alongside U.S. trainees. The number of U.S. institutions that have overcome the legal and financial barriers is small. Of note, U.S.-based trainees often provide direct patient care for short-term engagements abroad. Increasingly, LMICs are enforcing policies to ensure appropriate medical licensing policies and work visas apply to U.S. trainees on short-term rotations. Regardless, U.S.-based academic programs should provide oversight of trainees abroad and encourage participation in supervised clinical care alongside local LMIC partners. Financial challenges, such as a lack of funding for hosting trainees from LMIC settings, can only be addressed if there is institutional support for bidirectionality. Insights from this study may help identify areas where institutional advocacy and policy reform could be informed by partner priorities and lived experiences.

If costs are barriers to sustained bidirectionality, U.S.-based programs may consider optimizing technology when aligned with LMIC partner preferences and capacities [19]. Examples such as virtual didactics, free and open access modules or websites, virtual simulation, and shared platforms for telemedicine consultations or consultations over encrypted text messaging could be adopted. Some U.S. pediatric educators have created open-access websites and educational modules or podcasts that can be integrated into LMIC settings' educational curricula. Although virtual platforms can enhance bidirectionality, it is unclear whether LMIC partners prefer such modes of communication. Limitations were demonstrated during the COVID-19 pandemic, where virtual engagement was limited in scope and in its ability to make a meaningful, sustained impact [20]. Significant barriers such as a lack of technological infrastructure for internet connectivity, equipment, and online journal or resource access, in addition to time zone differences, can prohibit equity within GHPs [19,21]. Understanding the LMIC sites’ abilities, priorities, and preferences is critically necessary to inform how the HIC partners can work alongside them and how each organization can mutually benefit from this GHP.

U.S.-based faculty can also look into creative solutions to cover travel costs for trainees and faculty from LMICs, such as utilizing institutional stipends for grand rounds presentations at the U.S. program. Partnering amongst U.S. programs can help reduce the costs of travel for visiting trainees and faculty; for instance, a visitor from an LMIC may visit more than one program on a trip, dividing travel costs between programs. And although visitors from international partner sites to the U.S. often can only participate in observerships (with limited hands-on clinical practice), they can be encouraged to meaningfully participate and teach at pediatrics didactics, receive training on how to best utilize library resources, and participate in simulation curriculum [2]. Of note, there are a few programs that have been able to sponsor J1 visas for visitors, whereby visiting physicians are able to practice at the level of medical students. In addition, institutions from LMIC countries could cross-collaborate with similar clinical settings where the scope of practice is not limited and where there may be similar exposure to pathology and disease in a resource-limited setting. These approaches may serve as starting points for broader discussions with international partners regarding preferred models of exchange, scope of practice, and educational priorities.

When visitors from U.S.-based institutions travel abroad, they should be encouraged to understand the colonial history of GH and the power imbalance that exists between HIC and LMIC settings, despite best attempts to encourage bidirectionality. U.S.-based trainees should be active practitioners of anti-racism and equity work [22]. Furthermore, partners from HIC settings should acknowledge and mitigate differences in power and privilege. One tool that could be utilized in U.S.-based pediatric GH education is GHEARD (Global Health Education for Equity, Anti-Racism, and Decolonization), a modular curriculum for GH trainees and faculty designed to prompt important conversations around bias, discrimination, racism, systemic oppression, structural violence, and inequities [23]. In addition, U.S. trainees and faculty should be cognizant of burdening LMIC healthcare systems and of the short-term nature of their visits, with the aim of providing longitudinal, continuous, and frequent support if their LMIC partners desire it.

There are limitations to this study. Due to the survey being internally developed, there is a lack of validated survey items. Surveying only U.S.-based pediatric academic GH faculty and educators limits our understanding and does not take into account the broader perspectives of partners in LMIC settings or those working in non-academic settings in HIC. This is a starting point for future studies that will include the perspectives of LMIC partners in order to broaden our understanding of bidirectionality. Furthermore, we acknowledge that the majority of the U.S.-based academic GH faculty and educators in this study identify as white/Caucasian (15/22, 68%). This likely reflects the current demographics of U.S.-based pediatric academic GH faculty and educators and the need to further diversify the field [23]. The current findings are intended to inform, rather than substitute for, further inquiry into bidirectionality with international partners. In addition, free-text responses limited our ability to clarify certain pieces of data. For instance, four participants wrote that their programs did not have a GHP, and an additional two were unsure if they did. Interviews may be a better method for data collection on this topic. In addition, our response rate was 27%. Due to the subject matter of this study, it was likely that those willing to participate were more likely to have GHPs and be familiar with the term “bidirectionality.” Future studies to understand the perspectives of partners in LMIC settings regarding bidirectionality and GHP are imperative. Despite these limitations, we feel that the participants of the study contributed meaningful perspectives and insights into how U.S.-based pediatric GH faculty can approach and sustain equitable and respectful GHPs in the future.

Despite the many challenges present, participants indicated that they prefer to maintain their partnerships while adapting as they can to reach equitable and respectful bidirectionality in GHPs. However, these findings represent only one side of a complex, multilateral story; we must listen to LMIC partners directly affected by GHPs and allow them to guide us on what would be most meaningful and impactful. While mutual benefit and reciprocity are widely endorsed ideals, their actual realization and evaluation require active engagement with LMIC partners’ perspectives, priorities, and definitions of shared benefit [24]. Equitable practice in global health research suggests coordinated efforts to actively value and learn from LMIC partners’ lived experiences, priorities, and insights, rather than assuming a shared understanding of benefit or impact solely from the U.S. perspective [25]. More needs to be done, including in the present study, to engage in reciprocal dialogue with international partners that reflects the needs, values, and leadership of all involved partners. Our findings will be meaningfully and intentionally incorporated into future discussions with international colleagues on their own perspectives of bidirectionality.

This study is a first step in understanding bidirectionality from American global health practitioners. Although input from partners in Kenya was integral to this project, our hope is that in the next iteration, we seek the perspectives of our GH partners abroad on their thoughts about bidirectionality. We have shared the results of this project with global health colleagues and partners from abroad at the national conferences, and aim to have further discussions with our pediatric GH partners abroad about how to optimize bidirectionality based on local needs and mutual respect.

## Conclusions

Our results suggest that bidirectionality could be defined as a concept whereby two or more partners collaborate at the personal and institutional level, resulting in reciprocal and equitable exchange of benefits. Additional qualities may include mutual respect and understanding, not merely an exchange of financial or educational resources. Establishing bidirectionality in GHPs may look different across settings, but the mutual benefits, frequent reflections, and evaluations by both partners are necessary for successful bidirectionality to occur in GHPs. Future work should explicitly explore and center the perspectives of international partners to understand how bidirectionality is experienced within their local contexts, and to inform truly reciprocal bidirectionality in GHPs.

## Appendices

Appendix 1

Table 2 shows the study survey completed at enrollment.

**Table 2:** Study survey completed at enrollment

Question #	Question / Text	Response Options
—	“Thank you for agreeing to participate in this study. This survey will take approximately 15 minutes to complete. By proceeding to answer questions within this study, you are consenting to participation in it. Your answers will be confidential, and your name and program will not be associated with any data or findings that results from this study. Please know that there are no right or wrong answers to any of these questions. If you do not feel comfortable answering a question, you can click “prefer to not answer.” To thank you for your participation, we will email you a $5 Starbucks gift card upon completion of the survey. Please send any questions or concerns to Dr. Anik Patel at anik.patel.md@gmail.com”	—
—	We would like to start by getting to know you and your pediatric program.	—
1	What is your role at your pediatric program?	a. Global Health Direction; b. Global Health Educator; c. Faculty interested in global health; d. Faculty with no interest in global health; e. Fellow/Trainee; f. Other: _________________________; g. Prefer to not answer
2	Approximately how many categorical pediatric residents are at your pediatric program, per class/year?	____________
3	Approximately how many total pediatric residents are at your pediatric program, per class/year (including med/peds, triple board, EM/Peds, NDD, etc)?	____________
4	Does your program have a formal global health track?	a. Yes; b. No; c. Prefer to not answer
4.a.1	[If “Yes” to 4] Approximately how many pediatric residents participate in a global health track, per class?	____________
5	Does your program offer an elective in global health? (can check multiple items)	a. Yes. We offer a virtual/local global health elective; b. Yes. We offer international electives where trainees can travel to a site outside the U.S.; c. No; d. Prefer to not answer
6	Prior to the COVID-19 pandemic, approximately how many of these residents participated in a global health elective, per class?	____________
7	Approximately, what percentage of trainees at your program would identify global health as an area of interest?	a. 0-10%; b. 11-20%; c. 22-30%; d. 31-40%; e. 41-50%; f. >50%; g. Prefer to not answer
8	Does your pediatric program work closely with any global health sites?	a. Yes; b. No; c. Prefer to not answer
8.a.1	[if “yes” to 8] Please describe your pediatric program’s relationship with these sites.	[free text]
8.a.2	[if “yes” to 8] What is the term that you would use to describe these sites?	[free text]
—	We want to now explore the concept of “bidirectionality”	—
9	Are you familiar with the term “bidirectionality” in terms of global health?	a. Yes; b. No
9.a.1	[If “yes” to 9] What does the term “bidirectionality” mean to you?	[free text]
9.a.2	[If “yes” to 9] Are there any other ways that you’ve heard the term “bidirectionality” used within the global health context?	[free text]
—	Here are some examples of that others may describe as bidirectional within a global health setting:	o where trainees from one institution travel to the partner’s setting to learn from and teach each other, occurring in both directions; o where skilled personnel from one institution travel to the partner’s setting to learn from and teach others as well as provide clinical care; o where patients from one institution are able to be transferred to another institution for medical care; o where funding and resources from one institution are shared or given to the partnering institution to improve medical care/training; o where educational opportunities are shared between partnering institutions virtually as well as in person; o where medical providers from one institution are available to provide virtual consultations for those at another institution, and vice versa; o where programs partner together to perform research and create academic output together, each receiving equivalent credit
10	With the above examples (or your own) in mind, what are some potential benefits that can arise when bidirectionality is attempted or occurs?	[free text]
11	With the above examples (or your own) in mind, what are some potential challenges that can arise when bidirectionality is attempted or occurs?	[free text]
—	We would now like to understand more about your specific pediatric program in the context of global health and bidirectionality.	—
12	Do you believe your pediatric program is currently engaging in any aspect of bidirectionality?	a. Yes; b. No
12.a.1	[if “Yes” to 12] please describe how your program engages in bidirectionality?	[free text]
12.a.2	[if “Yes” to 12] what resources does your pediatric program draw from within your organization/community to make this possible?	[free text]
12.a.3	[if “Yes” to 12] what downsides or challenges have your pediatric program faced in engaging in a bidirectional partnership?	[free text]
12.a.4	[if “Yes” to 12] how has your pediatric program overcome these challenges, if applicable?	[free text]
12.a.5	[if “Yes” to 12] what are some benefits that your pediatric program obtains from a bidirectional partnership?	[free text]
12.a.6	[if “Yes” to 12] what are some benefits that your partnering program obtains from a bidirectional partnership?	[free text]
12.a.7	[if “Yes” to 12] what are some downsides or challenges that your partnering program may have engaging in a bidirectional partnership?	[free text]
13	Do you have examples of programs in other specialties at your institution that participate in bidirectional exchanges?	a. Yes; b. No
13.a.1	[if “Yes” to 13] Can you describe them for us and how they are bidirectional?	[free text]
13.a.2	[if “Yes” to 13] What works well for these programs?	[free text]
13.a.3	[if “Yes” to 13] What are some things that these programs don’t do as well?	[free text]
14	Do you have any other thoughts about global health partnerships or bidirectionality that you would like to share?	[free text]

Appendix 2

Table 3 shows the invitation email to potential participants

**Table 3:** Invitation email to potential participants and baseline survey provided

Section / Item	Text	Response Options
Outreach Email 1	Hello Dr. XXX,My name is [author name], and I am a pre-med student at [institution name]. My internship is conducting a research study pertaining to global health partnerships, and we are interested in learning more about your program and its views on those partnerships. I am wondering if you would be willing to take a brief survey and/or possibly participate in a quick interview in the future? Thank you for your time and consideration.Sincerely,XXX	—
Outreach Email 2	To whom it may concern:My name is [author name], and I am a pre-med student at [institution name]. I am working on a research study pertaining to global health partnerships and am wondering who the best person would be to contact to get more information about global health activities that your program offers. If your program offers global health programming, could you please share the contact information for the best person to contact about that?Thank you for your time and consideration.Sincerely,XXX	—
—	Demographics of Participant	—
Age	Age (in years):	a. <35; b. 36-45; c. 46-55; d. 56-65; e. >65
Race	Race (please select all that apply)	a. Black or African-American; b. American Indian, Native American, or Alaskan Native; c. Asian; d. Hispanic/Latinx; e. Native Hawaiian or Pacific Islander; f. White or Caucasian
Ethnicity	Ethnicity: Do you identify as Hispanic/Latinx?	a. Yes, I identify as Hispanic/Latinx; b. No, I do not identify as Hispanic/Latinx
Indigenous identity	Indigenous identity: Do you identify yourself as being of indigenous origin?	a. Yes; b. No
Gender	Gender: what is your gender identity?	a. Male; b. Female; c. Transgender (Male to Female); d. Transgender (Female to Male); e. Additional gender category (please specify):________________________; f. Prefer to not answer

## References

[1] Koplan JP, Bond TC, Merson MH, Reddy KS, Rodriguez MH, Sewankambo NK, Wasserheit JN (2009). Towards a common definition of global health. Lancet.

[2] Pitt MB, Gladding SP, Majinge CR, Butteris SM (2016). Making global health rotations a two-way street: A model for hosting international residents. Glob Pediatr Health.

[3] Butteris SM, Schubert CJ, Batra M (2015). Global health education in US pediatric residency programs. Pediatrics.

[4] Hau DK, Smart LR, DiPace JI, Peck RN (2017). Global health training among U.S. residency specialties: a systematic literature review. Med Educ Online.

[5] Drain PK, Holmes KK, Skeff KM, Hall TL, Gardner P (2009). Global health training and international clinical rotations during residency: current status, needs, and opportunities. Acad Med.

[6] Russ CM, Tran T, Silverman M, Palfrey J (2017). A study of global health elective outcomes: a pediatric residency experience. Glob Pediatr Health.

[7] Lu PM, Park EE, Rabin TL, Schwartz JI, Shearer LS, Siegler EL, Peck RN (2018). Impact of global health electives on US medical residents: a systematic review. Ann Glob Health.

[8] Bodnar BE, Claassen CW, Solomon J, Mayanja-Kizza H, Rastegar A (2015). The effect of a bidirectional exchange on faculty and institutional development in a global health collaboration. PLoS One.

[9] Keating EM, Haq H, Rees CA (2019). Reciprocity? International preceptors’ perceptions of global health elective learners at African sites. Ann Glob Health.

[10] Batra M, Pitt MB, St Clair NE, Butteris SM (2018). Global health and pediatric education: Opportunities and challenges. Adv Pediatr.

[11] Rohrbaugh R, Kellett A, Peluso MJ (2016). Bidirectional exchanges of medical students between institutional partners in global health clinical education programs: Putting ethical principles into practice. Ann Glob Health.

[12] Turissini M, Mercer T, Baenziger J (2020). Developing ethical and sustainable global health educational exchanges for clinical trainees: Implementation and lessons learned from the 30-year AMPATH partnership. Ann Glob Health.

[13] Hudspeth JC, Rabin TL, Dreifuss BA (2019). Reconfiguring a one-way street: A position paper on why and how to improve equity in global physician training. Acad Med.

[14] Seo SW, Ombengi D, Sultan DH (2020). An ethics-based approach to global health research part 1: Building partnerships in global health. Res Social Adm Pharm.

[15] Daffé ZN, Guillaume Y, Ivers LC (2021). Anti-racism and anti-colonialism praxis in global health—Reflection and action for practitioners in US academic medical centers. Am J Trop Med Hyg.

[16] Hussain M, Sadigh M, Sadigh M, Rastegar A, Sewankambo N (2023). Colonization and decolonization of global health: which way forward?. Glob Health Action.

[17] Kulesa J, Brantuo NA (2021). Barriers to decolonising educational partnerships in global health. BMJ Glob Health.

[18] Eichbaum QG, Adams LV, Evert J, Ho MJ, Semali IA, van Schalkwyk SC (2021). Decolonizing global health education: Rethinking institutional partnerships and approaches. Acad Med.

[19] Umphrey L, Lenhard N, Lam SK (2022). Virtual global health in graduate medical education: a systematic review. Int J Med Educ.

[20] McHenry MS, Tam RP, Nafiseh AA (2021). Global health partnerships during the COVID-19 pandemic: Perspectives and insights from international partners. Am J Trop Med Hyg.

[21] Umphrey L, Paasi G, Windsor W (2022). Perceived roles, benefits and barriers of virtual global health partnership initiatives: a cross-sectional exploratory study. Glob Health Res Policy.

[22] Tchonang Leuche V, Delgado-Zapata R, Umphrey L, Lam SK, Cardiel Nunez K, Musiime V, Rule A (2023). Decolonizing global child health education for more equitable and culturally safe collaborations. Pediatr Ann.

[23] Fanny SA, Tam RP, Rule A, Barnes A, Haq H (2024). Transforming pediatric global health education through antiracist and anticolonial principles. Pediatrics.

[24] Flynn E, Sommer A, Kapila P, Samuels H, Yennampelli S, Willis C, Meiring M (2026). Exploration of the effects of bidirectional learning in medical education among global health partnerships: A scoping review. Med Teach.

[25] Ingenhoff R, Sarker M, Hanson K (2025). Stronger together: advancing equity in global health research partnerships. BMJ Glob Health.

